# Grizzly Bear Noninvasive Genetic Tagging Surveys: Estimating the Magnitude of Missed Detections

**DOI:** 10.1371/journal.pone.0161055

**Published:** 2016-09-07

**Authors:** Jason T. Fisher, Nicole Heim, Sandra Code, John Paczkowski

**Affiliations:** 1 Alberta Innovates–Technology Futures, Ecosystems Management Unit. #3, 4476 Markham Street, Victoria, British Columbia, V8Z 7X8, Canada; 2 University of Victoria, School of Environmental Studies, PO Box 3060, STN CSC, Victoria, British Columbia, V8W 3R4, Canada; 3 Alberta Environment and Parks, Parks Division, Suite 201, 800 Railway Avenue, Canmore, Alberta, T1W 1P1, Canada; Smithsonian Conservation Biology Institute, UNITED STATES

## Abstract

Sound wildlife conservation decisions require sound information, and scientists increasingly rely on remotely collected data over large spatial scales, such as noninvasive genetic tagging (NGT). Grizzly bears (*Ursus arctos*), for example, are difficult to study at population scales except with noninvasive data, and NGT via hair trapping informs management over much of grizzly bears’ range. Considerable statistical effort has gone into estimating sources of heterogeneity, but detection error–arising when a visiting bear fails to leave a hair sample–has not been independently estimated. We used camera traps to survey grizzly bear occurrence at fixed hair traps and multi-method hierarchical occupancy models to estimate the probability that a visiting bear actually leaves a hair sample with viable DNA. We surveyed grizzly bears via hair trapping and camera trapping for 8 monthly surveys at 50 (2012) and 76 (2013) sites in the Rocky Mountains of Alberta, Canada. We used multi-method occupancy models to estimate site occupancy, probability of detection, and conditional occupancy at a hair trap. We tested the prediction that detection error in NGT studies could be induced by temporal variability within season, leading to underestimation of occupancy. NGT via hair trapping consistently underestimated grizzly bear occupancy at a site when compared to camera trapping. At best occupancy was underestimated by 50%; at worst, by 95%. Probability of false absence was reduced through successive surveys, but this mainly accounts for error imparted by movement among repeated surveys, not necessarily missed detections by extant bears. The implications of missed detections and biased occupancy estimates for density estimation–which form the crux of management plans–require consideration. We suggest hair-trap NGT studies should estimate and correct detection error using independent survey methods such as cameras, to ensure the reliability of the data upon which species management and conservation actions are based.

## Introduction

Biodiversity loss is one of the primary conservation concerns of the 21^st^ century [1–3]. Biodiversity loss is naturally the accumulation of declines of individual species, so single species-at-risk conservation is a major objective for many applied ecologists and wildlife management agencies [4, 5], especially in landscapes heavily impacted by anthropogenic landscape development [6]. Mammalian carnivores are particularly at risk. They are large and wide-ranging, have low reproductive rates, are sensitive to habitat fragmentation, and have been harvested or persecuted heavily since European colonization [7–9]. The need to detect and manage species declines has created a marked demand for inexpensive data collected over large spatial scales [10]. To meet this demand, mammal populations are often surveyed via noninvasive genetic tagging (NGT), which yields large volumes of inexpensive data on species’ occurrence [11]. Remotely collected hair samples can yield DNA, from which species, gender, individual, and genetic diversity can be ascertained through mitochondrial and microsatellite analysis [12, 13]. Noninvasive genetic tagging data inform (for example) estimates of population size and density [14–16], habitat selection [17], and landscape genetics–the landscape-scale analysis of population connectivity and gene flow [18].

Noninvasive genetic tagging has been used successfully for many mammal species [19], but few have been as intensely studied as grizzly bears, *Ursus arctos*. Grizzly bears have lost over half their historic North American range and remain only in the mountains and arctic of the northwest [9]. Grizzly bears have late primiparity and small, infrequent litters, and hence low reproductive potential [20–23]. Humans are a primary source of mortality [24, 25] with deaths spatially linked to road density and motorised access [26]. Therefore, habitat loss and human encroachment into core habitat are considered primary mechanisms of grizzly bear decline [27]. In Canada, the eastern edge of grizzly bears’ range sits in the province of Alberta, where they were listed as "Threatened" in 2010 with an estimated provincial-population size of 690 individuals [27]. This estimate is based largely on NGT data, which remains a primary source of information about spatiotemporal patterns in grizzly bear abundance in Alberta and across western North America (*e*.*g*. Kendall, Stetz [28]).

Genetic analysis for NGT has advanced markedly since its inception to overcome potential pitfalls [15, 29–31]. However, field sampling for NGT still carries several known and suspected sources of error. One notable problem with NGT surveys–as with many wildlife surveys–is that of false absences. When a species is not detected at a site, that species may be truly absent, or present but undetected, leading to biased estimates of occupancy and potentially false conclusions about distribution and abundance [32, 33]. Detection error is known to occur in NGT surveys [34]; methods to quantify and correct for this error are now being developed and explored, and have largely focussed on error imposed by capture heterogeneity and violation of closure assumptions [35–37].

Problems arise in NGT surveys when an animal visits a survey site but does not leave a hair sample with viable DNA; a species presence is counted as an absence. This can arise from temporal differences in hair retention by the pelt, sex- and age-specific rubbing behaviour [38], or DNA viability due to ambient temperature and moisture [39]. We advocate that the frequency of this detection error can be assessed using serial detection-nondetection data from unmarked animals (*e*.*g*. genetically identified to species, but not to individual) analysed in an occupancy framework [33]. Species occupancy (*ψ*)–the probability that a site is occupied by a species–is modelled in conjunction with its probability of detection (*p*)–the probability of detecting that species when it is present. As *p* is often less than one, naïve occurrence measures are negatively biased. A species’ probability of detection can vary among surveys, habitats, and seasons [33, 40]. Estimated *p* is also likely to vary among survey methods, since few methods are expected to sample the true population, and the efficacy of each method can differ [41]. For example, Fisher and Bradbury [42] showed that hair trapping could underestimate mustelid occurrence by as much as half, and that the presence of heterospecifics significantly affected hair capture probability. With research and management of species at risk relying so heavily on NGT data, the magnitude of missed hair detections is clearly important to assessing data reliability; yet to our knowledge few independent validations of detection error in NGT have been conducted.

We estimated detection bias for grizzly bear surveys by sampling the hair trap and the area around it with a second method: camera-trapping [43, 44]. Camera trapping is a popular technique for surveying mammals that yields a wealth of ecological information about species across large landscapes. Cameras can also assess detection bias in NGT surveys using multi-method occupancy models [41]. As *ψ* and *p* can differ among survey methods, employing multiple survey methods simultaneously allows researchers to estimate and account for bias in each method.

Our objective was to quantify NGT detection bias for grizzly bears on the East Slopes of Alberta’s Rocky Mountains, and to test predictions about temporal variability in detectability. We surveyed grizzly bear occurrence monthly between April and November 2012 and 2013 using cameras and hair trapping. We previously observed that (i) grizzly bears, like other species [38] display marked variation in their response to hair traps; (ii) pelts vary seasonally in their ability to retain hairs; and (iii) DNA viability varies with ambient temperature and moisture. Therefore we hypothesized (1) NGT underestimates detectability relative to cameras; (2) detection success via NGT varies through time, peaking in summer but declining in spring and fall; and (3) missed detections in NGT surveys are not fully accounted for in occupancy estimates by modelling *p*. The implications of these hypotheses are not trivial, as there remains no current consensus among grizzly bear researchers with regards to survey methods, survey duration, timing of sampling, and whether to use fixed traps or move them within a season. Variability in sampling design–such as short sampling periods or lumping surveys among months–may be entraining significant error, with ramifications for conservation and management decisions based on NGT surveys.

## Materials and Methods

### Study Area

**Fig 1:**
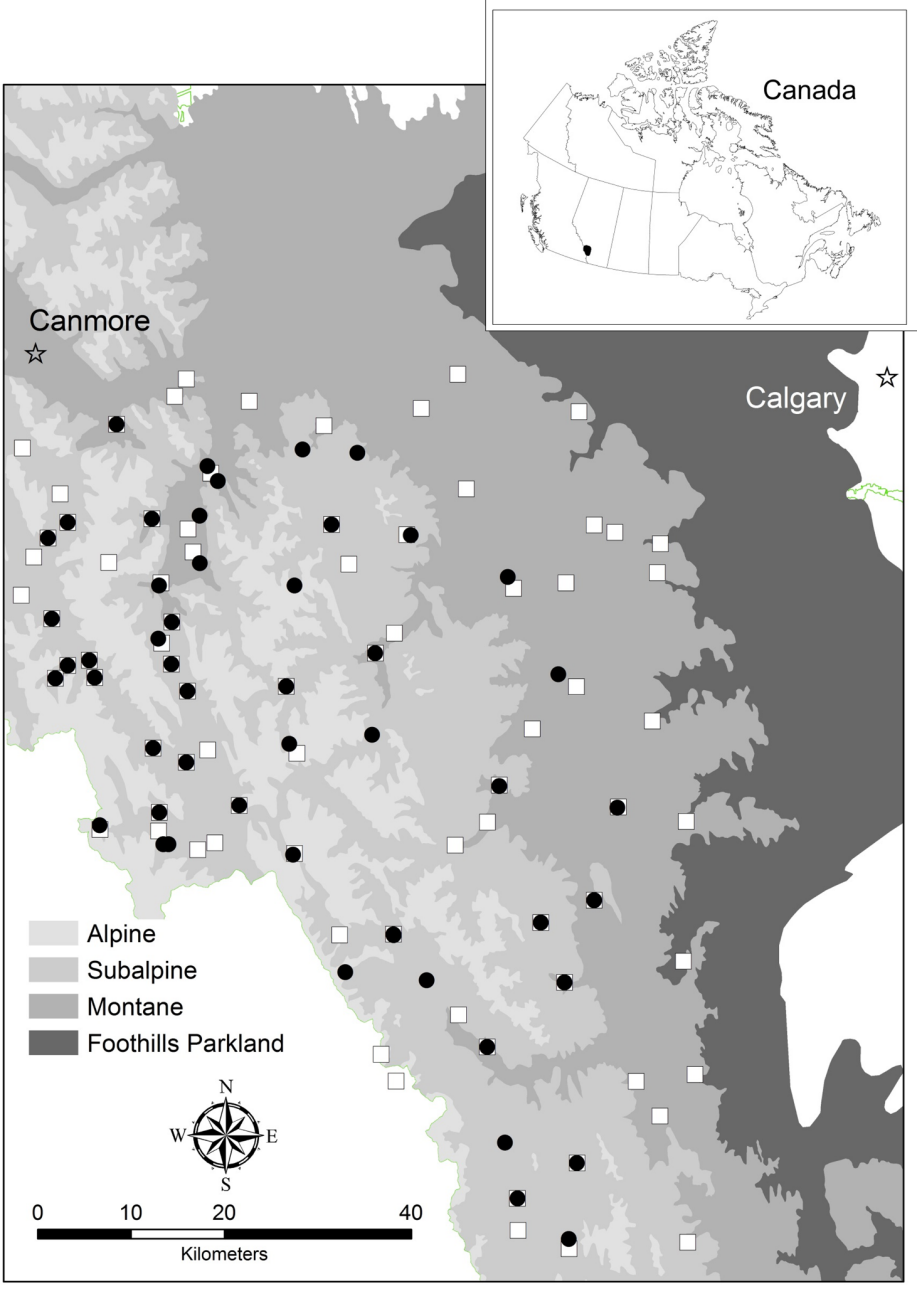
Grizzly bear occurrence sampling in the central Rocky Mountains and foothills of Alberta, Canada. Occurrence was surveyed at sampling sites in 2012 (black dots) and 2013 (white squares) deployed in a systematic design across alpine, subalpine, and montane ecoregions.

Grizzly bear distribution was sampled in the central Rocky Mountains of Alberta, Canada, within the Western Cordilleran system (Fig 1). The majority of this region sits within Alberta’s protected areas network, a landscape of varying degrees of legislated protection and intensive land-use, collectively termed Kananaskis Country. Some areas have only limited recreational development (e.g. hiking trails) whereas others are subject to forest harvesting, mining, petroleum extraction, transportation infrastructure, and hunting and trapping. Topography is rugged, with high peaks over 2500 m, steep-sloped ridges, and valley bottoms. Coniferous forest 80–120 years old (*Pinus contorta*, *Picea glauca*, *Picea mariana*, and *Abies balsamea*) dominate this landscape. Some small deciduous (*Populus tremuloides*, *Populus balsamifera*) stands occur throughout. Small stands of black spruce (*Picea mariana)* with forest floors dominated by Labrador tea (*Ledum groenlandicum*) and non-vascular plants occur in low-lying areas. Pine and mixed stands are often fairly open, with a sparse alder (*Alnus crispa*) understory. The area is home to a highly diverse mammal community [45] and bear food is considered abundant throughout the region.

### Study Design

We used a systematic sampling design consisting of 10-km x 10-km grid cells, plotted on a digital landscape coverage in ArcGIS (ArcGis 10.2 (Environmental Systems Research Institute, Inc., Redlands, CA, USA). Within each cell, we deployed a fixed sampling site which remained in place for the season. We subjectively deployed sampling sites generally at mid-elevation, in drainages or other travel corridors, with evidence of animal movement (Fig 1). Subjectivity at the site level maximizes probability of detection, but does not affect the probabilistic design as statistical inference is at the scale of the grid-cell. In some cells where known grizzly bear activity was concentrated, we divided the grid cell into 4 equal sections, and surveyed each of these smaller-scale grid cells to serve management objectives. Exploratory analysis showed detectability did not differ between these cells and the main grid so we pooled all sample sites. We surveyed 50 sites in 2012, and 76 sites in 2013, monthly between April (den emergence) and November (den re-entry). Specific sampling sites differed among years (to achieve other management objectives), so we analyzed each year's data separately.

### Species sampling

**Fig 2:**
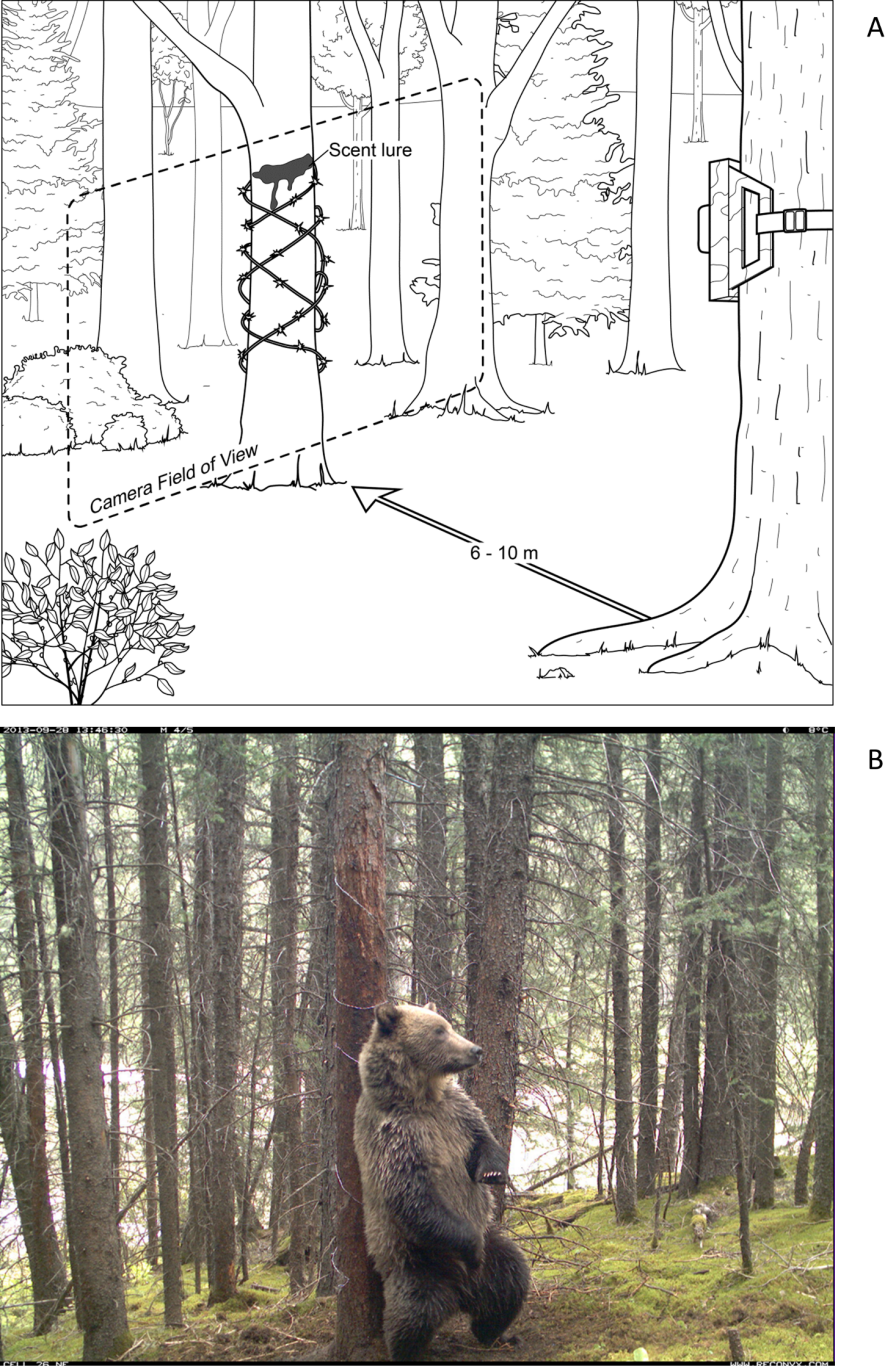
Double-method noninvasive sampling design for grizzly bears. Sampling sites consisted of a hair trap–a scent-lured tree wrapped with barbed wire–and a camera trap placed 6–10 m away to image the hair trap and the surrounding area (a). A grizzly bear encountering the trap could be imaged by the camera, but might not leave a hair sample with viable DNA (b).

We used two concurrent methods to sample grizzly bear occupancy: non-invasive genetic tagging (NGT) *via* hair sampling, and camera trapping (Fig 2A). Hair traps used Gaucho^®^ barbed wire (Bekaert, Brussels, Belgium) wrapped around a tree 2-m up the trunk. We smeared *ca*. 5 ml O’Gorman’s LDC Extra scent lure (O'Gorman's Co., Montana, USA) in patches on the trunk facing the camera. Grizzly bears investigating the tree rubbed and left hair samples with some degree of error, which we aimed to quantify (Fig 2B). We collected hair from the traps monthly, using sterile techniques. DNA from hairs was analysed by Wildlife Genetics International (WGI; Nelson, British Columbia, Canada) to identify species. DNA was extracted from hairs using QIAGEN®’s DNEasy™ Tissue Kits (QIAGEN, Hilden, Germany) and analysed to identify species using sequence-based analysis of the 16S rRNA gene of mitochondrial DNA (mtDNA) that was then compared against a DNA reference library of all known mammal species in the region.

At each station we also deployed one Reconyx™ RM30, PM30 or PC85 infrared-triggered digital camera (Reconyx, Holmen, Wisconsin, USA) *~* 6–10 m from the NGT hair-trap tree. Cameras detect species’ occurrence within an area defined by the size of their detection cone [46], and in our case includes both the hair trap and the area immediately surrounding that trap (Fig 2). Camera data were downloaded monthly in conjunction with hair data collection. Images were analysed and summarised for species presence within 30-day periods; each period constituted a single survey. Likewise, the hair collection during each 30-day period was considered as a single survey. The final data frame was comprised of 50 sites (2012) and 76 sites (2013), with 8 repeated monthly visits and 2 methods per site. Following our recommendations in Burton, Neilson [44], we define the study area as the region bounded by a minimum convex polygon around the edges of the camera array; the sampling unit as the systemic grid cell in which the camera is subjectively placed; and the sampling site as the 360° circle around the camera demarcated by the extent of the cameras' detection zone–approx. 2500 m^2^ –into which a passing grizzly may occur and be drawn to the lure to investigate.

### Ethics Statement

This research was conducted in part on public land in provincial protected areas. The Government of Alberta, Ministry of Environment and Parks–who also collected data as legal designated authority under The Wildlife Act–granted research permission. Landowner permission was sought and granted for all sites on private land. Field sampling protocols strictly conformed to Canadian Council on Animal Care (CCAC) Guidelines and were conducted with approval of Alberta Innovates–Technology Futures' (AITF) Animal Care and Use Committee, Protocol #2070M-A13/002/12-P01. All sampling procedures were reviewed and specifically approved as part of obtaining the government research permit. The noninvasive nature of sampling avoided distress to the designated "Threatened" grizzly bears we sampled.

### Statistical Analysis

We used the single-season, multi-method occupancy models of Nichols, Bailey [41] to estimate (i) the probability of grizzly bear occupancy at a site, (ii) the conditional probability that a present bear would leave a viable hair sample, and (iii) and the probability of detecting grizzly bears, if present at a site, within each year. These models assume that sites are closed changes in occupancy at the species level among years, or rather, that any such changes are non-Markovian (random) among sites and among surveys. For mobile animals, we assume that a species available for sampling has a non-zero probability of being present at the sample unit within the sampling period. Month-long surveys were designed to satisfy this condition, as grizzly bear is expected to traverse its home range in much less than a month. Though we use the term "occupancy" for consistency, for mobile animals occurrence at a site should be interpreted as "site use" rather than permanent residence [33, 47]. It is important to note that the definition of sampling units (or plot sizes) is an area of debate and ongoing research [44, 47] and so is the interpretation of conditional and large-scale occupancy parameters.

In our multi-method survey protocol, animals at the hair trap were fully exposed to the camera trap (Fig 2). The detection area of the cameras was greater than point-detection at the hair trap; barring camera failures (treated as missing data), there were no occasions where a bear was sampled at a hair trap without being sampled by a camera. This differs from the Nichols, Bailey [41] scenario wherein either device could fail to detect a species at a site. Here, one method (cameras) drives large-scale occupancy and the other method (NGT) is subset of those detections. Therefore NGT hair-traps were considered as the “immediate” sample location (*cf*. Nichols *et al*. 2008) and the combination of cameras and NGT traps as the larger-scale sample location, wherein:

*ψ* = Pr(sampling unit occupied);
*p*^s^_t_ = Pr(detection at survey *t* by method *s* | sample unit occupied and species present at immediate sample location);
*θ*_t_ = Pr(species occupying NGT site at occasion *t* | sample unit occupied);
ψ * θ_*t*_ = Pr(occupancy at NGT site)
*s* = 2 sampling devices, and *t* = 8 sampling occasions.

The conditional probability of occupancy *θ* is the parameter of particular interest here. It refers to the per-survey probability that a species occupying sampling sites will “occupy” a hair-trap, and so quantifies the degree of bias in NGT sampling, *sensu* Fisher and Bradbury [42]. We estimated *ψ*, *p*, and *θ* using multi-method hierarchical models in program PRESENCE ver. 9.3 [48], which employs maximum likelihood methods and generalised linear models to estimate parameters.

There are many possible causes of missed detections among surveys. Here, we explicitly acknowledge that probability of detection *p* is a function of *both* grizzly bear movement and missed detections at a sampling device. Consider for example a hair-trap detection history 101, which may arise from 2 processes. First, a bear may occur at a site in one month, but not the next, and then re-appear; in the '0' case the bear was present on its territory but moving about elsewhere rather than at our trap. Second, the bear may have been present at the site on all three occasions, but failed to leave a hair at the second occasion. In this case *p* conflates both the probability that a bear available for surveying does not appear at a site due to this vagility, as well as missed detections due to behaviour, environment, or sampling device failure. Multi-method occupancy models account for imperfect detection based on detection histories, but add the estimated parameter *θ*: a conditional probability of occupancy at one detection device, given probability of occupancy established by a combination of devices. An estimated *θ* < 1 at a hair trap is a function of hair from a photographed bear not being captured at a trap, or of DNA extraction failure from a captured hair sample. This key distinction between detectability and availability is not typically explicitly acknowledged in occupancy studies [47].

We constructed multiple competing single-season models to weigh the evidence in support of five hypotheses: detectability was either (1) constant, (2) differed between methods, (3) varied with each survey period, (4) varied as a trend through time, or (5) varied through time independently for each method. Conditional probability of occupancy *θ* was either constant or varied through time. Models were ranked using an information-theoretic approach based on Akaike’s Information Criterion (AIC) scores and their normalised AIC weights (AIC_w_), which describe the weight of evidence in support of each model [49]. We summed AIC_w_ and calculated evidence ratios (ER) for each model variable; ER = 2 suggests there is twice the evidence for inclusion of an explanatory variable than its exclusion. From per-survey estimates of *p* we calculated and plotted the probability of false absence (PFA) for a given survey duration as [1-*p*]^t^ [50] with t = 8 independent surveys. For comparison, we also constructed single-season single-method occupancy models for each device to compare detectability and occupancy estimates from each sampling approach, though Nichols, Bailey [41] explain why this is not advocated.

## Results

Grizzly bear detection was generally consistent among years. In 2012 we detected grizzly bears at 36/51 (70.5%) sites *via* cameras and at 29/51 (56.9%) of sites *via* hair traps. In 2013, we detected grizzly bears at 53/76 (69.7%) sites *via* cameras and at 43/76 (56.6%) of sites *via* hair traps. Grizzly bears occupied about three-quarters of sampling sites in 2012 (*ψ* = 0.77; s.e. = 0.07) and 2013 (*ψ* = 0.71; s.e. = 0.05) according to best-supported models (Table 1; AIC_w2012_ = 0.80; AIC_w2013_ = 0.99).

**Table 1:** Model selection of multi-method occupancy models of grizzly bears in the Rocky Mountains of Kananaskis Country, Alberta, Canada. Conditional probability of occupancy (*θ*) was either constant (.) or varied through time (t). Probability of detecting grizzly bears (*p*) was either constant (.), varied with METHOD, varied with each SURVEY, varied through time as a TREND, or varied INDEPENDENTly for each survey and method.

Model	AIC	ΔAIC	AIC_w_	Model likelihood	K*	-2LL**
*2012 sampling*						
ψ,θ(t),p(METHOD)	418.53	0.00	0.80	1.00	11.00	396.53
ψ,θ(t),p(TREND)	421.35	2.82	0.20	0.24	11.00	399.35
ψ,θ(t),p(.)	430.30	11.77	0.00	0.00	10.00	410.30
ψ,θ(.),p(INDEPENDENT)	434.86	16.33	0.00	0.00	18.00	398.86
ψ,θ(t),p(INDEPENDENT)	437.10	18.57	0.00	0.00	25.00	387.10
ψ,θ(.),p(METHOD)	439.02	20.49	0.00	0.00	4.00	431.02
ψ,θ(t),p(SURVEY)	440.17	21.64	0.00	0.00	17.00	406.17
ψ,θ(.),p(SURVEY)	440.95	22.42	0.00	0.00	10.00	420.95
ψ,θ(.),p(TREND)	447.61	29.08	0.00	0.00	4.00	439.61
ψ,θ(.),p(.)	452.07	33.54	0.00	0.00	3.00	446.07
*2013 sampling*						
ψ,θ(t),p(METHOD)	749.58	0.00	1.00	1.00	11.00	727.58
ψ,θ(.),p(METHOD)	763.07	13.49	0.00	0.00	4.00	755.07
ψ,θ(t),p(INDEPENDENT)	769.12	19.54	0.00	0.00	25.00	719.12
ψ,θ(t),p(.)	770.30	20.72	0.00	0.00	10.00	750.30
ψ,θ(t),p(TREND)	770.85	21.27	0.00	0.00	11.00	748.85
ψ,θ(t),p(SURVEY)	776.52	26.94	0.00	0.00	17.00	742.52
ψ,θ(.),p(INDEPENDENT)	780.84	31.26	0.00	0.00	18.00	744.84
ψ,θ(.),p(TREND)	784.68	35.10	0.00	0.00	4.00	776.68
ψ,θ(.),p(.)	785.38	35.80	0.00	0.00	3.00	779.38
ψ,θ(.),p(SURVEY)	789.55	39.97	0.00	0.00	10.00	769.55

*number of parameters in the model

**-2 log likelihood of the model (deviance)

**Fig 3:**
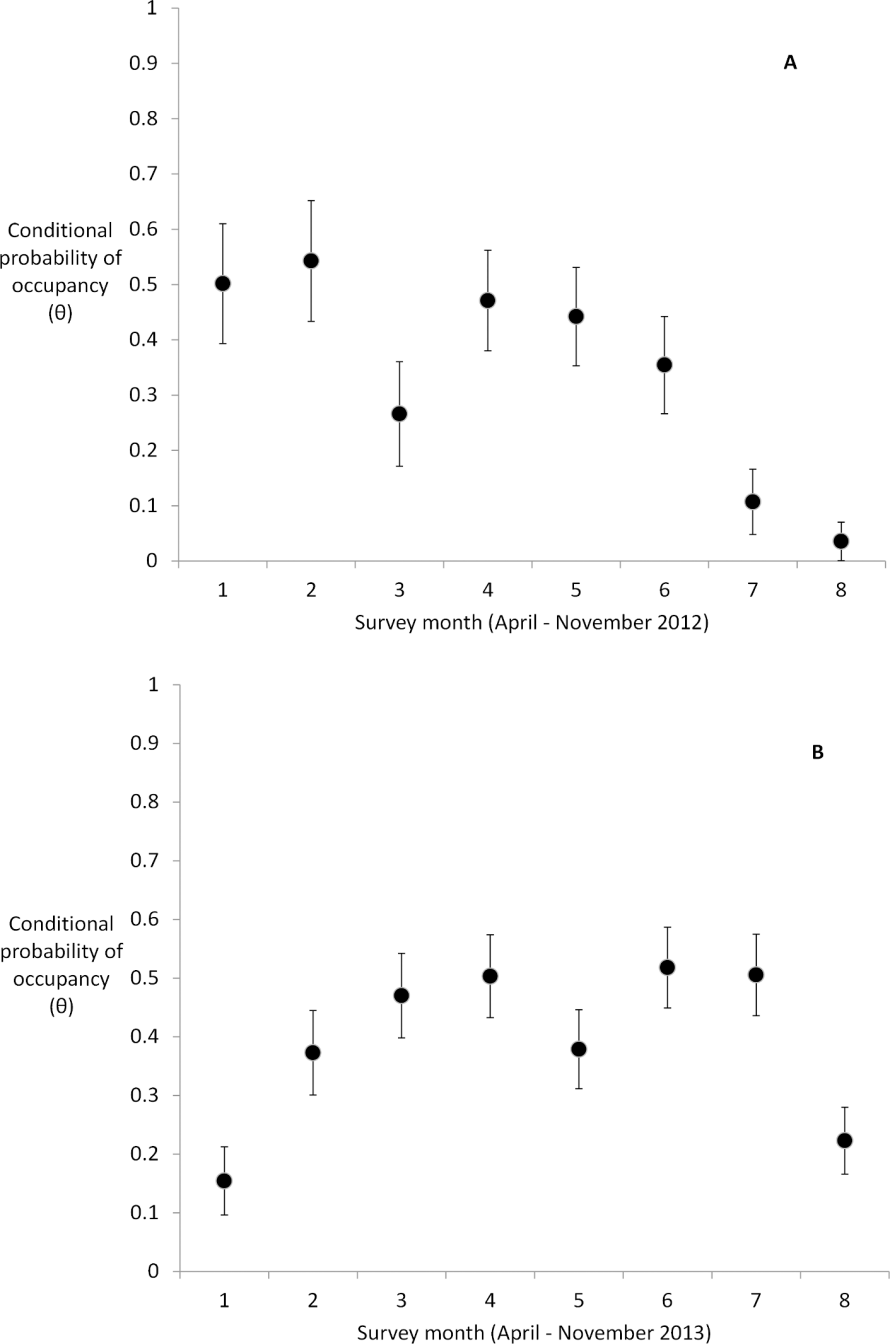
Conditional probability of grizzly bear occupancy at a hair trap, given occupancy as evidenced by combined methods. Conditional occupancy varied differently among months in (a) 2012 and (b) 2013.

The conditional probability that a grizzly would “occupy” a hair trap–given that its presence was confirmed by cameras–varied with each monthly survey in 2012 (ER_θt_ = 832) and 2013 (ER_θt_ = 3332) (Fig 3). Grizzly bears were most likely to occupy hair traps in spring and summer in 2012, and in summer in 2013. In 2012, conditional occupancy at the scale of the hair trap was at best 0.55 (s.e. = 0.11), and at worst 0.03 (s.e. = 0.03), depending on survey month. In 2013, conditional occupancy ranged from 0.52–0.15 (s.e. = 0.07, 0.06). Notably, there was a brief reduction in conditional occupancy at the hair trap in mid-summer in both years, roughly occurring in June 2012 and August 2013.

**Fig 4:**
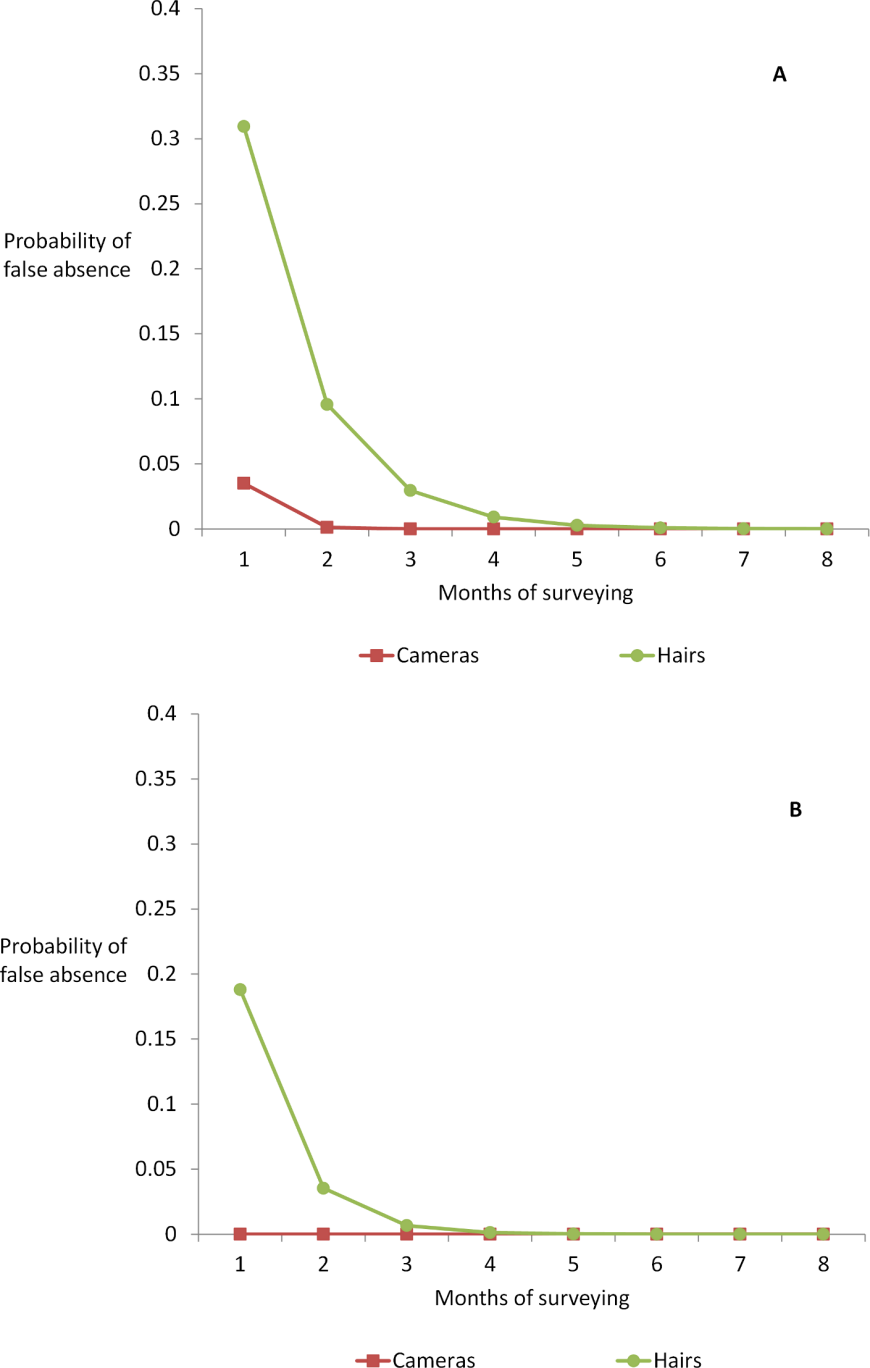
Probability of false absence (PFA) of grizzly bears at camera traps and hair traps. PFA is 1-p (per survey probability of detection), compounded monthly, in (a) 2012 and (b) 2013.

Hair traps were less likely to detect grizzly bears than were camera traps. The top model in both years suggests that probability of detection varies by method (ER_method2012_ = 4; ER_method2013_ = 9999; Table 1). Cameras reliably detected grizzly bears when present (p_2012_ = 0.96; p_2013_ ~ 1.0). Hair traps were less likely to detect a grizzly bear if present (p_2012_ = 0.69; p_2013_ = 0.81). These are per-survey estimates; when compounded through time the probability of false absence declines (Fig 4). After three monthly surveys there is a less than 0.05 probability of false absences from NGT hair-trapping; this approaches zero after four months.

Single-season, single-method occupancy models corroborate our findings. Best supported models suggest *p* varies among surveys for hair traps (AIC_w2012 =_ 0.97, *p*_2012_ = 0.04–0.54; AIC_w2013 =_ 0.99, *p*_2013_ = 0.18–0.61). The same was true of camera traps (AIC_w2012 =_ 1.0, *p*_2012_ = 0.00–0.52; AIC_w2013 =_ 0.99, *p*_2013_ = 0.17–0.47). Most notably, occupancy estimated via hair traps was always lower than for camera traps in both 2012 (ψ_camera_ = 0.77, se = 0.07; ψ_hair_ = 0.66, se = 0.08) and in 2013 (ψ_camera_ = 0.72, se = 0.05; ψ_hair_ = 0.63, se = 0.06).

## Discussion

Genetic data are remarkably valuable for identifying individuals, mapping distribution, estimating density, assessing relatedness, and investigating gene flow through landscape genetics–provided that biases in genetic analysis [11] and in the detection process [42] can be modelled and accounted for. We show that independent validation of NGT-based sampling via cameras reveals sometimes substantial detection bias in this important mode of ecological inquiry. Unmodelled heterogeneity in detection (hence capture) rates can violate the assumptions of statistical models using NGT data, such as density estimation models [51–53]. If sampling design–specifically, the timing and duration of sampling–imparts sampling error by sampling for too short a duration, or moving sites under the assumption that all sampling periods provide equal detectability, then resulting density estimates may be biased, with implications for conservation decisions relying on those data.

We found that monthly hair-trap NGT surveys underestimated grizzly bear occupancy by a widely fluctuating margin, depending on the month. Variability in *p* was in part a result of survey-to-survey differences in the rate at which species appeared at a trap–as many past studies have acknowledged, even if not explicitly [17, 42, 44, 54, 55]. Partitioning variance between *θ* and *p* yielded little evidence that *p* varied among surveys, suggesting comparatively less temporal heterogeneity was imparted by grizzly bear movement–a conclusion reached by Rovang, Nielsen [55] in their occupancy analysis of grizzly bears in an area north of ours.

Critically, the source of error assumed in *p* is different than the source of error in *θ* estimated by the multi-state occupancy model. Estimated *θ* can be interpreted as probability of occupying the hair trap, given large-scale occupancy, and is subject to variability both by grizzly bear movement (modelled as *p*) and by the efficacy of the hair trap relative to the cameras. The difference in detection error is presumably due to variability in bears' willingness to rub on the hair trap (Fig 2), the degree to which pelts retain or release hairs, or the decay rate of DNA in hair samples due to ambient temperature and moisture [14, 39, 56]. Co-occurrence of other species at the hair trap can also reduce or facilitate hair deposition [42]. Most likely, *θ* < 1 results from a combination of these factors, and Efford and Dawson [47] discuss these at length. The mechanisms require further examination, but regardless, we demonstrate that this rate of error can be substantial and varies through time. Estimated *θ* shows sampling success is not equal among months, as concluded by Rovang, Nielsen [55]. The timing of sampling matters. This fact can impart significant error if sampling sites are moved around but pooled and analysed as a single season, a natural design choice when seeking to maximum sampling sites *n* [57–61], but with unknown consequences. On the other hand, repeated monthly sampling can reduce this error to negligible margins, which is fine for occupancy studies; but density models are heavily influenced by per-survey detections to estimate numbers of unknown individuals, so missed detections may influence these estimates to an unknown degree.

It is important to note that occupancy modelling is not a panacea to the problem of detection error; models are based on multiple assumptions that may (or may not) be met in any given repeat-sampling design [47, 62], and camera trapping is a special subset of this question [44]. Occupancy models do offer an explicit framework for formulating and testing hypotheses about process errors. We also note that although we used monthly samples, weekly (or any other temporal schedule) could be used, and this will change estimated *p* for mobile animals as *p* depends greatly on the frequency of site use. Finally note that although the wrapped-tree sampling method is gaining popularity it differs from the "wire corral" typically used in grizzly bear NGT surveys [63]. Corrals rely on a hair capture from a bear as it enters or exits to get bait. Our lured-tree method stimulates a repeated rub response (Fig 2), thus multiplying chances for a hair capture (but also potentially entraining error from age-sex differences in rubbing behaviour). Our probability of detection was *p* = 0.7 (2012) and *p* = 0.8 (2013), greater than values typically reported for corral traps [55, 64]. Repeating monthly surveys four times reduces this error to near zero. Moreover, *p* at cameras was 0.97–1.0, providing a vital independent validation of the rates of missed detections. The extent of genetic sampling underestimation cannot be known without camera traps; such independent validation and multi-state modelling provides an empirical lens through which to view the accuracy of NGT estimates.

## Conclusions

Missed detections are a non-trivial problem inherent in all surveys, and we show that missed detections in NGT hair-trapping surveys can bias occupancy estimates markedly, and through time. Reliable scientific inference requires that survey methods measure and account for this heterogeneity. First, we recommend that grizzly bear NGT surveys should both maximize probability of detection (*p*) and accommodate within-season changes in conditional occupancy at a hair trap (*θ*). NGT surveys should be conducted for a minimum of 3 repeated monthly surveys at fixed sampling points, and aim for four surveys where logistics permit. Fewer surveys results in high probabilities of false absence (missing grizzlies where they do occur), and risk negatively biasing occupancy estimates. Second, NGT surveys should be conducted when the probability that a visiting bear will leave viable DNA is the greatest. Over the summer, conditional occupancy *θ* fluctuates, and repeat surveys buffer against this detection error. Finally, NGT surveys should be validated with camera surveys. Camera data are vital in quantifying the bias associated with hair sampling, and correcting for this bias. Further, cameras provide data on reproductive success across space [54] and behaviour at the hair trap [42], data not available from NGT surveying alone. Cameras need not be deployed at every survey station, but should be deployed at a random subsample of survey sites.

A final question remains: How do missed detections translate into potentially biased abundance and density estimates? A great deal of effort has gone into understanding sources of heterogeneity in bear NGT surveys, and this is an ongoing area of research [35–37, 65–67]. We suggest that density models be subject to a sensitivity analysis, wherein random samples are dropped as missed detections to determine how missed hairs translate into missed bears. With ecological inference and conservation actions relying so heavily on NGT surveys for bears and many other species, understanding the consequences of detection error is vital to making effective conservation and management decisions.

## Supporting Information

S1 Table Grizzly bear detection histories in the Kananaskis Country of Alberta, Canada. For each survey session (month) 1–8, a bear was either detected (1) or undetected (0) at a hair trap (H) and a camera trap (C).
(XLSX)
